# *QuickStats:* Percentage of Emergency Department Visits for Pain* at Which Opioids^†^ Were Given or Prescribed, by Patient Age and Year — National Hospital Ambulatory Medical Care Survey, United States, 2010–2020

**DOI:** 10.15585/mmwr.mm715152a4

**Published:** 2022-12-30

**Figure:**
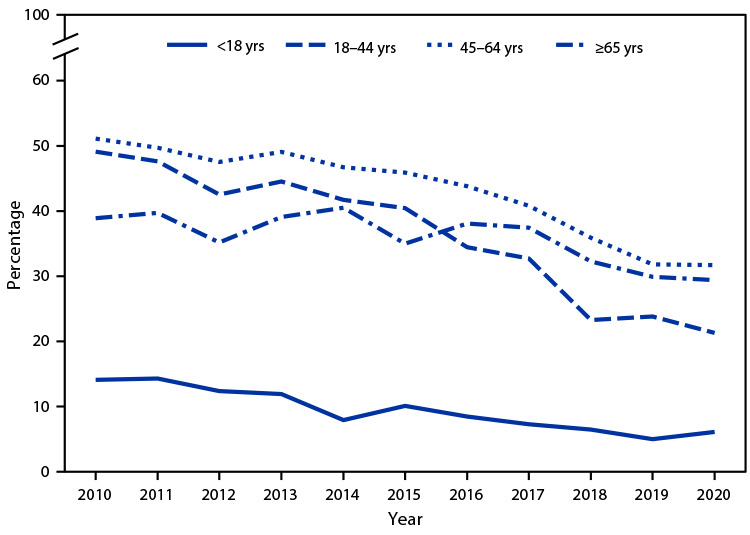
During 2010–2020, the percentages of ED visits for pain in which an opioid was given or prescribed decreased for all age groups. During this period, visits were lowest for persons aged <18 years, decreasing from 14.1% in 2010 to 6.1% in 2020. Among the adult age groups, adults aged 18–44 years experienced the greatest decrease during the period, declining from 49.1% to 21.3%. At the beginning of the period, percentages were lower for adults aged ≥65 years compared with those aged 18–44 years, but in 2016 that pattern reversed.

**Source:** National Center for Health Statistics. National Hospital Ambulatory Medical Care Survey, 2010–2020. https://www.cdc.gov/nchs/ahcd/index.htm

For more information on this topic, CDC recommends the following link: https://www.cdc.gov/drugoverdose/index.html

